# Resistomycin attenuates triple-negative breast cancer progression by inhibiting E3 ligase Pellino-1 and inducing SNAIL/SLUG degradation

**DOI:** 10.1038/s41392-020-00255-y

**Published:** 2020-07-29

**Authors:** Shan-shan Liu, Jie Qi, Zu-dong Teng, Fu-tao Tian, Xiao-xi Lv, Ke Li, Ya-jie Song, Wei-dong Xie, Zhuo-wei Hu, Xia Li

**Affiliations:** 1 Molecular Immunology and Pharmacology Group, State Key Laboratory of Bioactive Substance and Function of Natural Medicines, Institute of Materia Medica, Chinese Academy of Medical Sciences & Peking Union Medical College, Beijing, 100050 China; 2 Department of Pharmacy, Marine College, Shandong University, Weihai, 264209 China; 3 Department of Pathology, Changle People’s Hospital, Weifang, 262499 China; 4 Institute of Medicinal Biotechnology, Chinese Academy of Medical Sciences & Peking Union Medical College, Beijing, 100050 China

**Keywords:** Target identification, Breast cancer

**Dear Editor,**

The epithelial-to-mesenchymal transition (EMT) is a positive modulator of triple-negative breast cancer (TNBC) progression.^1^ Several EMT-inducing transcriptional factors (EMT-TFs) drive EMT process and enhance TNBC progression.^2^ Promoting the degradation of EMT-TFs to inhibit EMT and malignant actions holds therapeutic potential for TNBC patients.

Pellino-1, an E3 ubiquitin ligase, has been reported to contribute to lymphoid and several solid tumorigenesis.^3,4^ However, it is not clear whether Pellino-1 exerts a crucial role in the promotion of TNBC. We found that Pellino-1 was upregulated in tumor tissues compared with adjacent non-tumor tissues (Table S1, Fig. 1a and Supplementary Fig. S1a). Interestingly, higher Pellino-1 expression was found in TNBC patients than those in luminal and Her2^+^ subtypes. Also, elevated Pellino-1 was expressed in breast cancer cells than in normal cells (Supplementary Fig. S1b). Immunohistochemistry of the breast cancer tissue microarrays (TMAs) corroborated our finding that Pellino-1 expression was higher in TNBC relative to other subtypes (Supplementary Fig. S1c). In addition, the TMAs analysis indicated a negative correlation of high Pellino-1 expression with TNBC survival (Fig. 1b). Silencing *PELI1* suppressed migration, invasion, and tumorsphere formation both in primary TNBC cells and in MDA-MB-231 cells (Supplementary Fig. S1d–g). Moreover, Pellino-1 depletion in PDX-1 and MDA-MB-231 suppressed tumor progression in mice, as indicated by the reductions in tumor weight and metastasis (Fig. 1c, Supplementary Fig. S1h, i), and the elevation in the survival rates of tumor-bearing mice (Supplementary Fig. S1j). These data indicate that high Pellino-1 expression promotes TNBC progression.

**Fig. 1:**
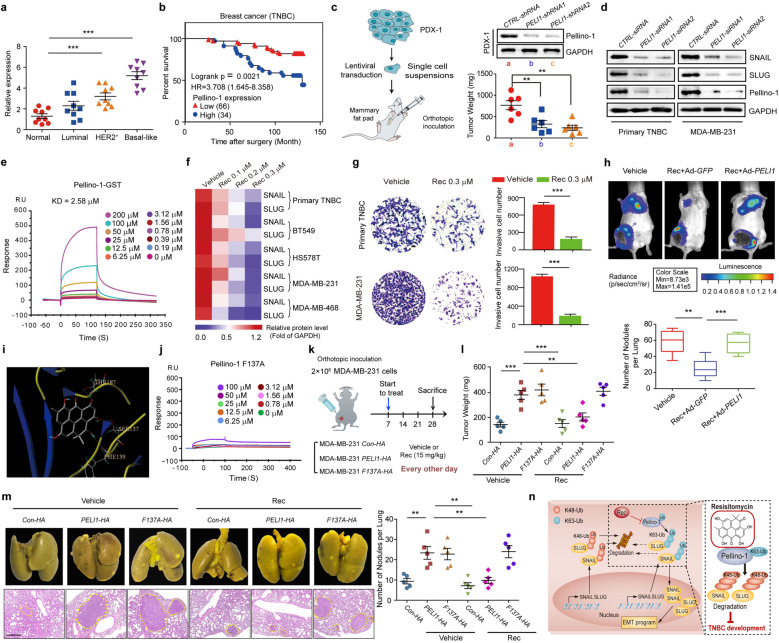
**a** Pellino-1 expression was detected by western blotting in tumor and adjacent non-tumor tissues of human breast cancer (normal *n* = 9, luminal *n* = 9, HER2^+^*n* = 9, and TNBC *n* = 9). **b** Kaplan–Meier plot showed overall survival of TNBC patients in tissue microarray sections (HBreD140Su03) depending on the expression of Pellino-1 (*n* = 100). **c** The strategy for studying the effects of Pellino-1 in patient-derived tumor xenograft (PDX) model from TNBC patients (*n* = 6 per group) and statistical analyses of the tumor weights in the PDX model after Pellino-1 knockdown. **d** Immunoblots showing the expression of SNAIL and SLUG after Pellino-1 knockdown (*n* = 3). **e** The indicated concentrations of Resistomycin were passed over immobilized Pellino-1-GST on CM5 sensor chips and the kinetic interaction of Resistomycin with Pellino-1 was determined with SPR analyses (*n* = 3). **f** Heat map presenting the expression levels of SNAIL and SLUG in 4 TNBC cell lines and 1 primary human TNBC cells after indicated concentrations of Resistomycin treatment (*n* = 3). **g** Primary TNBC cells and MDA-MB-231 cells were treated with Resistomycin for 24 h, and the transwell assay was applied to assess invasion (*n* = 3). The number of invading cells was calculated in three different fields. Scale bar, 20 μm. **h** MDA-MB-231 cells were injected into the mammary fat pad of nude mice unilaterally. One week later, mice were treated with Vehicle or Resistomycin, and infected with Ad-*GFP* or Ad-*PELI1* for 3 weeks (*n* = 6 per group). Data are bioluminescence imaging of mice (top) and statistical analyses of lung metastatic nodules (down). **i** The binding mode of Resistomycin to the homology model of the FHA domain by using crystal structure of Pellino-2 (PDB code 3EGA) as the template. Pellino-2 is shown in ribbon plot representation. Compounds are labeled in color by atoms. The hydrogen bonds are labeled as dashed lines. The key amino acid residues for the binding are labeled as sticks. **j** SPR analyses of the kinetic interaction of Resistomycin and Pellino-1 F137A (*n* = 3). **k** The strategy for studying the anti-tumor effects of Resistomycin in the presence or absence of Pellino-1 or Pellino-1-F137A overexpression in vivo. **l**, **m** The indicated MDA-MB-231 cells were injected into the mammary fat pad of nude mice unilaterally. One week later, mice were treated with Vehicle or Resistomycin for 3 weeks (*n* = 5 per group). Data are statistical analyses of the tumor weights (**l**), macroscopic and histopathologic images of lung metastatic nodules and statistical analyses of these nodules (**m**). Scale bar, 200 μm. **n** Schematic diagram illustrates the role of Resistomycin in inhibition of TNBC progression through promoting SNAIL/SLUG degradation via binding with Pellino-1. *P < 0.05, **P < 0.01, ***P < 0.001

Consistent with the study reported in lung cancer cells,^3^ Pellino-1 knockdown decreased the expression of SNAIL and SLUG in primary TNBC and MDA-MB-231 cells (Fig. 1d). Depletion of Pellino-1 did not change the mRNA expression, but reduced the K63-linked and increased the K48-linked polyubiquitinated forms of SNAIL and SLUG (Supplementary Fig. S2a–c). Consequently, silencing Pellino-1 shortened the degradation half-lives of SNAIL and SLUG (Supplementary Fig. S2d). Positive correlations between the expression of Pellino-1, SNAIL, and SLUG were found in TNBC samples (Supplementary Fig. S2e). Notably, patients with higher expression of Pellino-1 and SNAIL/SLUG had a lower survival rate than others (Supplementary Fig. S2f). Furthermore, overexpression of SNAIL and SLUG abrogated the effect of Pellino-1 knockdown on the enhanced *E-cadherin* expression and on the suppressed migration and invasion of MDA-MB-231 cells (Supplementary Fig. S2g–j). Consistent with the in vitro analysis, overexpression of SNAIL or SLUG reversed the reductions in tumor weight and lung metastasis (Supplementary Fig. S2k–m) caused by Pellino-1 depletion. These results indicate that silencing Pellino-1 expression suppresses TNBC progression via the downregulation of SNAIL and SLUG expression.

We thus investigated why silencing Pellino-1 increased the SNAIL/SLUG degradation. We found that Pellino-1 depletion increased the interactions between SNAIL/SLUG and FBXO11 (Supplementary Fig. S3a), which is the only E3 ubiquitin ligase mediating the degradation of SNAIL and SLUG simultaneously. Indeed, knockdown of Pellino-1 could not reduce the expression of SNAIL and SLUG in FBXO11-silenced cells (Supplementary Fig. S3b). These data indicate that the reduction of SNAIL and SLUG levels following Pellino-1 depletion are mediated by the E3 ligase FBXO11.

Following the procedures outlined in Supplementary Fig. S3c, we made effort to identify potential inhibitors of Pellino-1. Among this library which contains 354 compounds, only 19 increased the E-cadherin luciferase activity by more than 2 fold (Supplementary Fig. S3d). A second round screening confirmed that only 2 compounds decreased the protein level of SNAIL and SLUG simultaneously (Supplementary Fig. S3e). However, only one compound, Resistomycin,^5^ disrupted the interaction between Pellino-1 with SNAIL/SLUG (Supplementary Fig. S3f, g). Resistomycin displayed a high affinity for GST-Pellino-1 (KD = 2.58 μM) as indicated by surface plasmon resonance (Fig. 1e). Whereas, there was no interaction between Resistomycin and GST (Supplementary Fig. S3h). Indeed, Resistomycin reduced the association of Pellino-1 and SNAIL/SLUG (Supplementary Fig. S3i), but enhanced the association of FBXO11 and SNAIL/ SLUG (Supplementary Fig. S3j).

We next investigated the action mechanism of Resistomycin in TNBC cells. Resistomycin treatment reduced the expression of SNAIL and SLUG in TNBC cells (Fig. 1f). In addition, Resistomycin exposure shortened the half-lives of SNAIL and SLUG degradation (Supplementary Fig. S4a, b). Resistomycin had no effect on total Ub, but decreased the K63-linked polyubiquitinated forms and increased the K48 polyubiquitinated forms of SNAIL and SLUG (Supplementary Fig. S4c, d). Overexpression of Pellino-1 reversed the downregulation of K63-linked polyubiquitinated forms and the upregulation of K48-linked polyubiquitinated forms of SNAIL and SLUG caused by Resistomycin (Supplementary Fig. S4e, f), suggesting that Resistomycin reduces the K63-linked and increases the K48-linked polyubiquitination of SNAIL and SLUG through Pellino-1. Furthermore, Resistomycin treatment not only changed the cell morphology from a spindle-shape into squamous, a typical morphology for epithelial changed cells, but also led to the increase in E-cadherin expression and the decreases in N-cadherin and Vimentin expression (Supplementary Fig. S4g, h). Then we evaluated the effect of Resistomycin on TNBC malignancy. Cell migration, invasion, and tumorsphere formation were impeded in cells treated with Resistomycin (Fig.1g, Supplementary Fig. S5a-c). Administration of Resistomycin dose-dependently decreased tumor growth and weight in PDX models (Supplementary Fig. S5d–g). Importantly, Resistomycin exposure showed no obvious toxic effects in normal mice, as indicated by survival rate and renal/liver functions (Supplementary Fig. S6a–c).

We then examined if the anti-tumor effects of Resistomycin were mediated by Pellino-1. We found that overexpression of Pellino-1 not only abrogated the effect of Resistomycin on the expression of EMT markers, but also reversed the suppressed migration, invasion, tumor growth and metastasis caused by Resistomycin treatment (Supplementary Fig. S6d–i, Fig. 1h). The high sequence similarity of Pellino-2 with Pellino-1 suggested that Resistomycin might bind to Pellino-2 and suppress its function. Indeed, Resistomycin decreased LPS-enhanced mRNA level of *IL-1β* (Supplementary Fig. S6j), which is mediated by Pellino-2 and suggests that Resistomycin suppresses Pellino-2 function. However, Pellino-2 depletion showed no effect on the expression of SNAIL/SLUG, proliferation, and invasion of MDA-MB-231 cells (Supplementary Fig. S6k–m). Moreover, silencing Pellino-3 also did not affect the invasion of MDA-MB-231 cells (Supplementary Fig. S6n). These data indicate that Resistomycin attenuates TNBC progression via the specific inhibition of Pellino-1 function.

The homology modeling approach and molecular docking suggested that Resistomycin formed hydrogen bonds with Ser137, Phe139, and Thr187 in Pellino-2 which are homologous to Ser135, Phe137 and Thr185 in Pellino-1 (Fig.1i, Supplementary Fig. S6o). To determine the amino acid responsible for the Resistomycin/Pellino-1 interaction, we constructed these point mutations individually or simultaneously. Resistomycin lost the ability to suppress SNAIL/SLUG expression only in F137A–overexpressed cells (Supplementary Fig. S6p). And no interaction between Resistomycin and GST-Pellino-1 F137A was found (Fig. 1j). Furthermore, Resistomycin treatment only reversed the enhanced tumor weight and metastasis caused by WT Pellino-1 but not by F137A overexpression (Supplementary Fig. S6q, Fig. 1k–m). Additionally, Resistomycin only suppressed EMT caused by WT Pellino-1, but not F137A (Supplementary Fig. S6r). Overall, these data suggest that Resistomycin inhibits TNBC progression by binding to the F137 of Pellino-1 to interfere with Pellino-1 function.

In summary, our study not only reveals a positive correlation of Pellino-1 expression with TNBC progression, but also shows that Resistomycin can bind with Pellino-1, interrupt the interaction of Pellino-1 with SNAIL/SLUG, and attenuate EMT in TNBC (Fig. 1n). Thus, Resistomycin holds the promise for further development as a novel inhibitor of Pellino-1 for antagonizing the invasive TNBC progression.

## Supplementary information

Supplementary Information

## Data Availability

The data that support the findings of this study are available from the lead corresponding author (X.L.) on reasonable request.
