# Supplementary material for: Resistomycin attenuates triple-negative breast cancer progression by inhibiting E3 ligase Pellino-1 and inducing SNAIL/SLUG degradation
Source: Signal Transduct Target Ther. 2020 Jul 29;5:133. doi: 10.1038/s41392-020-00255-y (PMC7391765; doi:10.1038/s41392-020-00255-y)
Supplement: Supplementary file 1 — Supplementary Information [file 41392_2020_255_MOESM1_ESM.docx]

Supplementary Materials for

Resistomycin Attenuates Triple-Negative Breast Cancer Progression by Inhibiting E3 Ligase Pellino-1 and Inducing SNAIL/SLUG Degradation

Shan-shan Liu^2^*, Jie Qi^1^*, Zu-dong Teng^3^, Fu-tao Tian^3^, Xiao-xi Lv^2^, Ke Li^4^, Ya-jie Song^1^, Wei-dong Xie^1^, Zhuo-wei Hu^2#^ and Xia Li^1#^

^1^Department of Pharmacy, Marine College, Shandong University, Weihai 264209, China

^2^Molecular Immunology and Pharmacology Group, State Key Laboratory of Bioactive Substance and Function of Natural Medicines, Institute of Materia Medica, Chinese Academy of Medical Sciences & Peking Union Medical College, Beijing 100050, China

^3^Department of Pathology, Changle People’s Hospital, Weifang 262499, China

^4^Institute of Medicinal Biotechnology, Chinese Academy of Medical Sciences & Peking Union Medical College, Beijing 100050, China

*These authors contributed equally to this work.

^#^Correspondence to: [xiali@sdu.edu.cn](file:///I:\QJ%20课题\V13\xiali@sdu.edu.cn); [huzhuowei@imm.ac.cn](mailto:huzhuowei@imm.ac.cn)

**This PDF file includes:**

Materials and Methods

Supplementary Text

Figures. S1 to S6

Tables S1 to S2

**Materials and Methods**

**Human cancer specimens**

Breast cancer tissues were obtained from Anyang Tumor Hospital, Henan University of Science and Technology and Changle People’s Hospital after surgical resection. Informed consent was obtained from all participants in accordance with the Declaration of Helsinki. All protocols using human specimens were approved by the Institutional Review Board of the Chinese Academy of Medical Sciences and Peking Union Medical College. The clinical features of the patients are list in Table S1 and S2.

**Tissue microarray**

Paired cancer and adjacent non-cancer paraffin tissue sections (BC081120b) were purchased from Alenabio (Xian, China). Pellino-1 was immunostained and scored as negative (0), weak (1), moderate (2) or strong (3) according to staining intensities. Samples were classified into binary groups either of low (scores 0 and 1) or high (scores 2 and 3) expression. Breast cancer paraffin tissue sections from patients with follow-up visit (HBreD140Su03) were purchased from Shanghai Outdo Biotech (Shanghai, China).

**Animal Studies**

Six weeks old Female athymic nude mice (Vital River Lab Animal Technology Co., Ltd) and NSG (NOD-Prkdcscid Il2rgnull) (Model Animal Resource Information Platform) were maintained in animal facility at the Institute of Materia Medica under Specific Pathogen Free (SPF) conditions. Both were housed in maximum barrier facilities, with individually ventilated cages, sterilized food and water. For animal studies, mice were ear marked before grouping and then were randomly separated into groups by an independent person. All animal procedures were conducted in accordance with the guidelines of the Institutional Committee for the Ethics of Animal Care and Treatment in Biomedical Research of Chinese Academy of Medical Sciences and Peking Union Medical College (Permit No. 002802).

Human patient-derived xenografts (PDX) primary cells were isolatedby using Tumor Dissociation Kit human (Miltenyi Biotec GmbH, CA, USA). Afterwards, they were spin-infected with virus-containing media supplemented with 2 mg/mL polybrene for 2 h at 1000 g at 4°C and then transplanted into the 4th mammary fat pad of 6-week-old female NPG mice.

To measure the antimetastatic effect of Resistomycin, one week after tumor inoculation, when transplanted cells had established, the mice were treated with the Resistomycin (7.5 mg/kg or 15 mg/kg) or Doxorubicin (2 mg/kg) every other day for 3 weeks. Six animals in each group were estimated that would give enough power without statistical analysis. All animals were included for final data analyses without any exclusion. Metastatic tumors were counted in a genotype-blinded manner under dissection scope. To evaluate survival rate, these mice were monitored for 80 days.

**Generation of PDX animal models**

Fresh breast cancer tissues were spliced into small fragments (1–3 mm^3^) in the medium. The tissue fragments were suspended in diluted Matrigel (Corning, 354248) 1:1 with PBS, and subcutaneously implanted into NPG mice (NOD-Prkdcscid Il2rgnull, Beijing Vitalstar Biotechnology). Early passages (1–5) of primary tumor tissues from these PDX models were mechanically minced and dissociated using gentleMACSTM Dissociator (Miltenyi Biotec) in accordance to the manufacturer’s protocols.

**Cell Culture**

The immortalized human mammary epithelial cells (HMLE) were provided by Dr.Guangli Suo (Suzhou Institute of Nano-Tech and Nano-Bionics (SINANO), Chinese Academy of Sciences). The human mammary epithelial cells (MCF10A) and human breast carcinoma cell lines (MCF-7, HS578T, MDA-MB-468, MDA-MB-231 and BT549) were purchased from the Shanghai Institute for Biological Sciences (SIBS), Chinese Academy of Sciences, where they were recently authenticated by STR profiling, and characterized by mycoplasma detection and cell vitality detection. And they were cultured in RPMI-1640 medium (Hyclone) and DMEM (Hyclone) containing 10% fetal bovine serum (FBS) at 37 ^o^C in a humidified atmosphere of 5% CO_2_. Primary human breast cancer cells were purchased from Cell Biologics (Chicago, USA) and maintained in primary cell culture system under 5% CO_2_.

**Generation of Stable Cell Lines**

To generate cells stably expressing *Control*^cas9^ and *PELI1*^cas9^, *PELI1*^Cas9^ lentiviral particles were purchased from GenScript Inc., and the gRNA sequence targeting Pellino-1 was 5’-AGCATAAATCCGTGCTGTAA-3’. Stable transfectants were selected by puromycin. Afterwards, Con-HA, Pellino-1-HA and Pellino-1 F137A plasmids were transfected into the above cells with Lipofectamine 3000 tansfection reagent (Invitrogen) according to the manufacturer’s instructions. After 24 h of transfection, stable transfectants were selected in medium containing 200 mg/mL hygromycin (Calbiochem, San Diego, CA, USA) for 7 days. After two or three passages in the presence of hygromycin, the cultures were used for experiments without cloning. To generate cells stably expressing *CTRL-shRNA*, *PELI1-shRNA1* and *PELI1-shRNA2*, those lentiviral particles were purchased from Beijing likely biotechnology Inc. Stable transfectants were selected by puromycin.

**siRNA sequences**

The RNAi sequences against human PELI1 and PELI2 were purchased from Guangzhou RIBOBIO Co., Ltd. Silencing *PELI1/2* was achieved by targeting the sequences 5’-CCUGGAAGUCAGAGUAAUUTT-3’ (*PELI1- siRNA1*), 5’-CCUGGA AUAUGGAGAGAAATT-3’ (*PELI1-siRNA2*), 5’-GTGTACACCTTGCGAGAAA- 3’(*PELI2-siRNA1*), 5’-GCCAACTCATGCTTTCACT-3’ (*PELI2-siRNA2*). The RNAi sequences 5’-CCCAGTTTCAGACACTCAT-3’ (*PELI3-siRNA1*) and 5’-GGATGGA CTGACCACCAAT-3’ (*PELI3-siRNA2*) against human PELI3 were purchased from Beijing likely biotechnology Inc. Non-sense control RNAi was also purchased from Beijing likely biotechnology Inc, and used as RNAi-negative control. Transient transfections were performed on 6-well plates using Lipofectamine® RNAiMAX reagent (Thermo Fisher, Beijing, China) according to the manufacturer’s instructions.

**Transwell invasion assay**

Transwell chambers (Merck Millipore, Darmstadt, Germany) were precoated with Fibronectin (10 μg/mL) on the lower surface, and the polycarbonate filter was coated with Matrigel (30 mg per well; BD Matrigel Matrix). Then, 2×10^5^ cells were placed on the upper layer in a serum-free culture medium. The lower chambers were placed with culture medium containing 10% FBS. After 24 h, cells on the upper side of the filter were removed with a cotton swab. The invaded cells were fixed with 4% paraformaldehyde, stained with crystal violet staining solution and counted under brightfield microscopy at × 200 in six random fields.

**Migration assay**

Confluent cell monolayers treated with Resistomycin (0.2 μM) were wounded by manually scraping the cells with a pipette tip, washed with PBS and further cultured in medium supplemented with 0.4% fetal bovine serum (FBS) for indicated time. Images were captured at 0, 24, 36 and 48 h after wounding with an Olympus CKX41 microscope, and the lesion area was measured (medium alone containing 0.4% FBS were used as control).

**Tumorsphere Assays**

For tumorsphere assays, PDX cells were seeded on 96-well ultralow attachment plates (Corning, NY, USA) at a density of 500 cells/well in StemXVivo Serum-Free Tumorsphere Media (R&D, Minnesota, USA) in the presence of indicated treatment. Images were acquired by using phase contrast microscope (Olympus Microsystems, CA, USA) and spheres were counted at 5-7 days later. For MDA-MB-231 cells tumorsphere assay, 250 μL cell/fibrinogen mixtures was made by mixing the same volume of the fibrinogen (Sea Run Holdings, Maine, USA) and cell solution (104 cells/mL), and seeded into 24 well-plate and mixed well with pre-added 5 μL thrombin (0.1 U/μL) (Sea Run Holdings). Then 1 mL complete medium was added after incubation for 10 min at 37°C cell culture incubator and spheres were counted at 4-7 days later.

**Luciferase reporter assay**

The E-cad-GLuc (HPRM12692-PG04) vector was obtained from GeneCopoeia, Inc. Cells were seeded on 12-well plates and transfected with the indicated plasmids. 24 h later, the cells were treated with various concentrations of Resistomycin and incubated for another 24 h. Then the culture supernatants were collected. The luciferase activity was measured with the luciferase assay kit according to the manufacturer’s instruction (GeneCopoeia).

**Western blot**

Proteins were extracted from cells or lung tissues using RIPA buffer (Cell Signaling Technology), and the protein concentrations were determined with BCA Protein Assay Kit. Protein sample were separated using SDS-PAGE, transferred to a PVDF membrane, and subjected to immunoblot analysis using specific antibody obtained from Cell Signaling Technology (Danvers, MA), including Vimentin (5741S), E-cadherin (14472S), SNAIL (3879S), SLUG (9585S), TWIST (46702S), ZEB1 (70512S), Pellino-1 (31474S), GAPDH (5274S) and N-cadherin (13116S). Binding of the primary antibody was detected by peroxidase-conjugated secondary antibodies and enhanced chemiluminescence. The signaling was captured by a LAS4000 Image Station (General Electric Company, Fairfield, CT, USA).

**RNA Extraction and Quantitative real-time polymerase chain reaction (RT-PCR)**

Total RNA was extracted using Trizol Reagent (Invitrogen) and the cDNA was synthesized by reverse transcription using M-MLV Reverse Transcriptase and Oligo (dT) primers (Promega). qPCR was performed using the KAPA SYBR FAST qPCR Master Mix (2×) Kit (Kappa Biosystem, USA) according to the manufacturer’s instructions with detection system of 95 °C for 3 min, 60 °C for 30 s and 95 °C for 5 s, and 35 cycles. The primers (5′ to 3′) for RT-PCR were listed as follows: E-cadherin Forward: 5′-TACG CCTGGGACTCCACCTA-3′, Reverse: 5′-CCAGAAACGGAGG CCTGAT-3′. GAPDH: 5′-GAGTCAACGGATTTGGTCGT-3′, Reverse: 5′-TTGATT TTGGAGGGATCTCG-3′. The mRNA expression levels were calculated by the 2^−ΔCt^ [Δ Ct = Ct (targeting gene)-Ct (GAPDH)] threshold cycle method.

**Immunofluorescence staining**

The cells were cultured on sterile cover slips, fixed with 4% paraformaldehyde for 15 min and permeabilized with 0.1% Triton X-100 for 15 min. Then, the cells were blocked with 3% bovine serum albumin (BSA) for 60 min at room time and incubated with primary antibody against E-cadherin (1:100, CST) and Vimentin (1:200, CST) at 4°C overnight. Specific binding of primary antibodies was detected using corresponding secondary antibodies. Nuclei were counterstained with DAPI. Images were captured using a confocal fluorescent microscope (Olympus Microsystems, CA, USA).

**Immunoprecipitation**

HEK293T cells were transfected with the indicated plasmids and were cultured for 24 h. The cells were collected and lysed in 550 μL Co-IP lysis buffer including 50 mM Tris (pH 7.5), 150 mM NaCl, 5 mg/mL aprotinin, 1 mg/mL pepstatin, 1% Nonidet P-40, 1 mM EDTA and 0.25% deoxycholate for 30 min. After centrifuge for 30 min at 12000 rpm, 10% of soluble lysates were divided as input, the remainder was incubated with specific antibody at 4°C overnight, followed by incubation with Protein A/G Plus–Agarose (Santa Cruz) beads at 4°C for 2 h. Then the immunocomplexes were mixed with 5x loading buffer and boiled for 10 min at 98°C. The precipitated proteins were subjected to SDS-PAGE gel and analyzed with corresponding antibody.

**Plasmid construction**

The site-directed mutants of Pellino-1 (S135A, F137A, T185A, S135A/F137A, F137/T185A, S135A/T185A, S135A/F137A/T185A). Ubiquitin (Ub), Ub^K48^, and Ub^K63^ sequences were synthesized commercially and constructed into pFLAG-CMV2 vector to establish the Ub-DDK, Ub^K48^-DDK and Ub^K63^-DDK expressing plasmids. SNAIL-Myc and SLUG-Myc plasmids were purchased from Sino Biological Inc (Beijing, China).

**Cycloheximide treatment**

To determine protein degradation, cells were incubated with the protein synthesis inhibitor cycloheximide (CHX, 10 μg/mL) for the indicated times and the expression of indicated proteins were evaluated by immunoblotting and quantitative analyses.

**Analysis of surface plasmon resonance**

Binding kinetics between GST, Pellino-1-GST, or Pellino-1 F137A-GST and Resistomycin were measured by surface plasmon resonance using a BIAcore T200 instrument (GE Healthcare, Pittsburgh, USA). The dissociation constant (KD) was calculated according to the BIA-evaluation software. Langmuir module was used to determine the KD for Resistomycin.

**Tumor Organoids Culture**

Fresh primary human tumors were rinsed with antibiotic wash, minced, and then digested in collagenase. After differential centrifugation at 1,500 rpm × 4–6 spins, tumor organoids were separated out, seeded to either Matrigel or 3D collagen I, and cultured in human mammary epithelium medium containing insulin, EGF, hydrocortisone, and cholera toxin. Medium was replaced every 3 to 4 days. The number of invasive cell numbers per organoids (>100 mm in diameter) was counted from 7–14 days after seeding.

**Statistical analysis**

Data are presented as the mean ± standard error of the mean (SEM). Student’s t test (2-tailed) was used to compare difference between two groups. One-way ANOVA with Tukey-Kramer’s comparison test was used to analyze difference among multiple groups. Generally, all assays were carried out with n ≥ 3 biological replicates. P < 0.05 was considered statistically significant. Analyses were performed using Graphpad Prism 6.0 software.

**Supplementary Text**

**Authors’ contributions**

X.L. and Z.H. raised conceptions and participated in the overall design, supervision and coordination of the study. S.L. and J.Q. designed and performed most of experiments. Z.T. and F.T. followed up patients and collected clinical information and materials. X.L. participated in molecular and cellular biological experiments. K.L. performed animal studies. W.X. and Y.S. extracted and purified the compound. Z.H., X.L., and S.L. wrote the manuscript. All authors read and approved the manuscript.

**Figure. S1.**

**
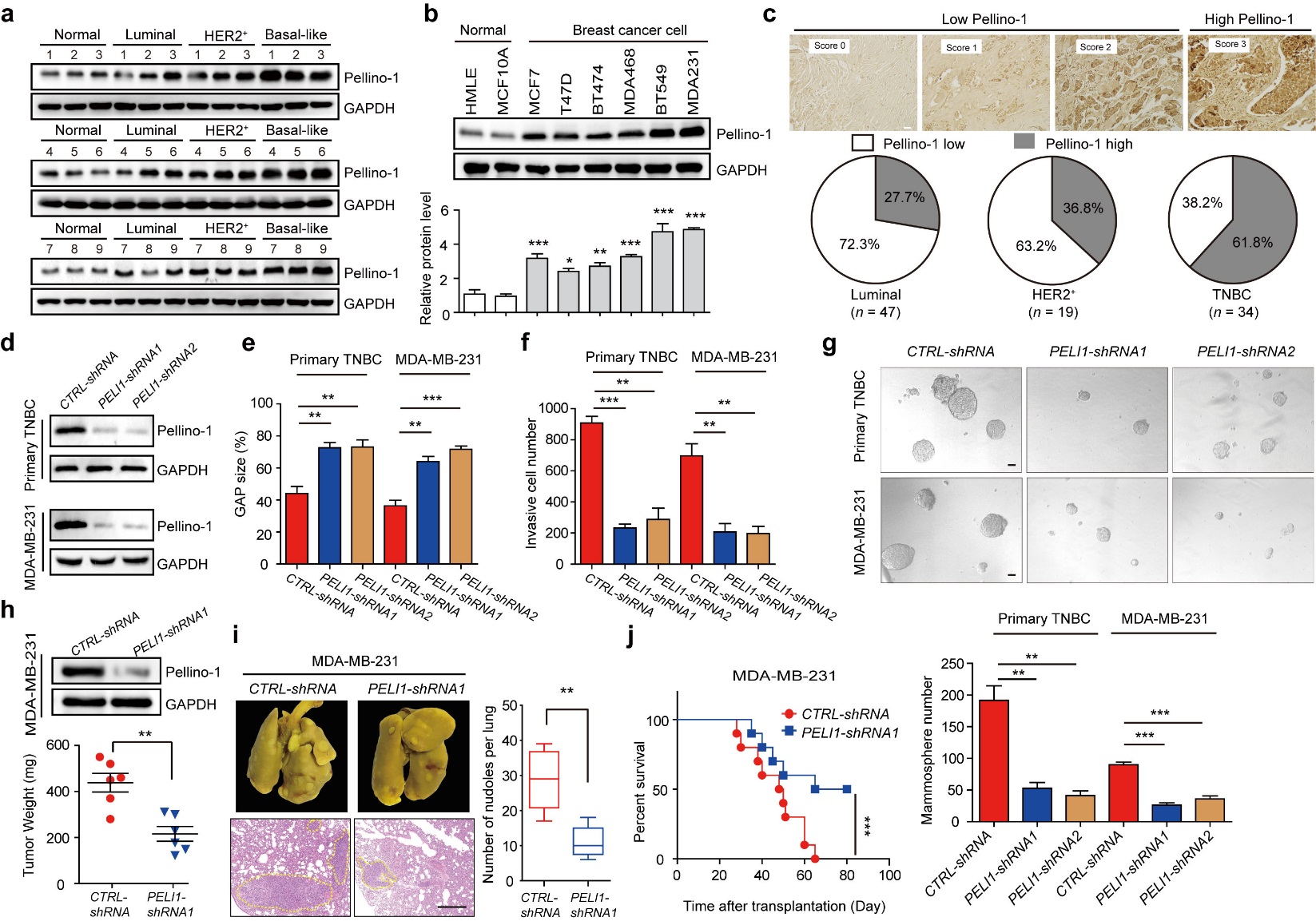
**

**Figure S1. Pellino-1 expression is positively associated with TNBC malignancy. a** Sample immunoblots of Pellino-1 expression in tumor and adjacent non-tumor tissues of human breast cancer (Normal n = 9, luminal n = 9, HER2^+^ n = 9, and TNBC n = 9). **b** Immunoblots of protein lysates from HMLE, MCF10A and breast cancer cells (n = 3). **c** Immunohistochemical (IHC) staining against the Pellino-1 protein in Formalin- ﬁxed, parafﬁn-embedded tissue microarray sections of breast cancer patients (BC081120b). After scoring Pellino-1 expression in each tissue, expression level was analyzed according to breast cancer subtypes (luminal n = 47, HER2^+^ n = 19, and TNBC n = 34 patients). Scale bar, 200 μm. **d** Expression of Pellino-1 was detected by western blotting in *PELI1*-*shRNA1/2* versus *CTRL*-*shRNA* cells (n = 3). **e** Silencing *PELI1* inhibited the migration of primary TNBC cells and MDA-MB-231 cells (n = 3). **f** Cell invasion was suppressed both in *PELI1*-silenced primary TNBC cells and MDA-MB-231 cells (n = 3). **g** Silencing *PELI1* inhibited tumor spheroid formation of primary TNBC cells and MDA-MB-231 cells. Data were showed as number of mammospheres per 1000 cells (n = 3). Scale bar, 50 μm. **h, i** MDA-MB-231 cells transfected with either *CTRL-shRNA* or *PELI1-shRNA1* were injected into mammary fat pad of nude mice unilaterally for 4 weeks (n = 6 per group). Data are statistical analyses of tumor weights (h), histopathologic images of lung metastatic nodules and statistical analyses of lung metastatic nodules (i) of indicated group. Scale bar, 200 μm. **j** Survival rates were analyzed by the Kaplan-Meier method (n = 10). *P < 0.05, **P < 0.01, ***P < 0.001.

**Figure. S2.**

**
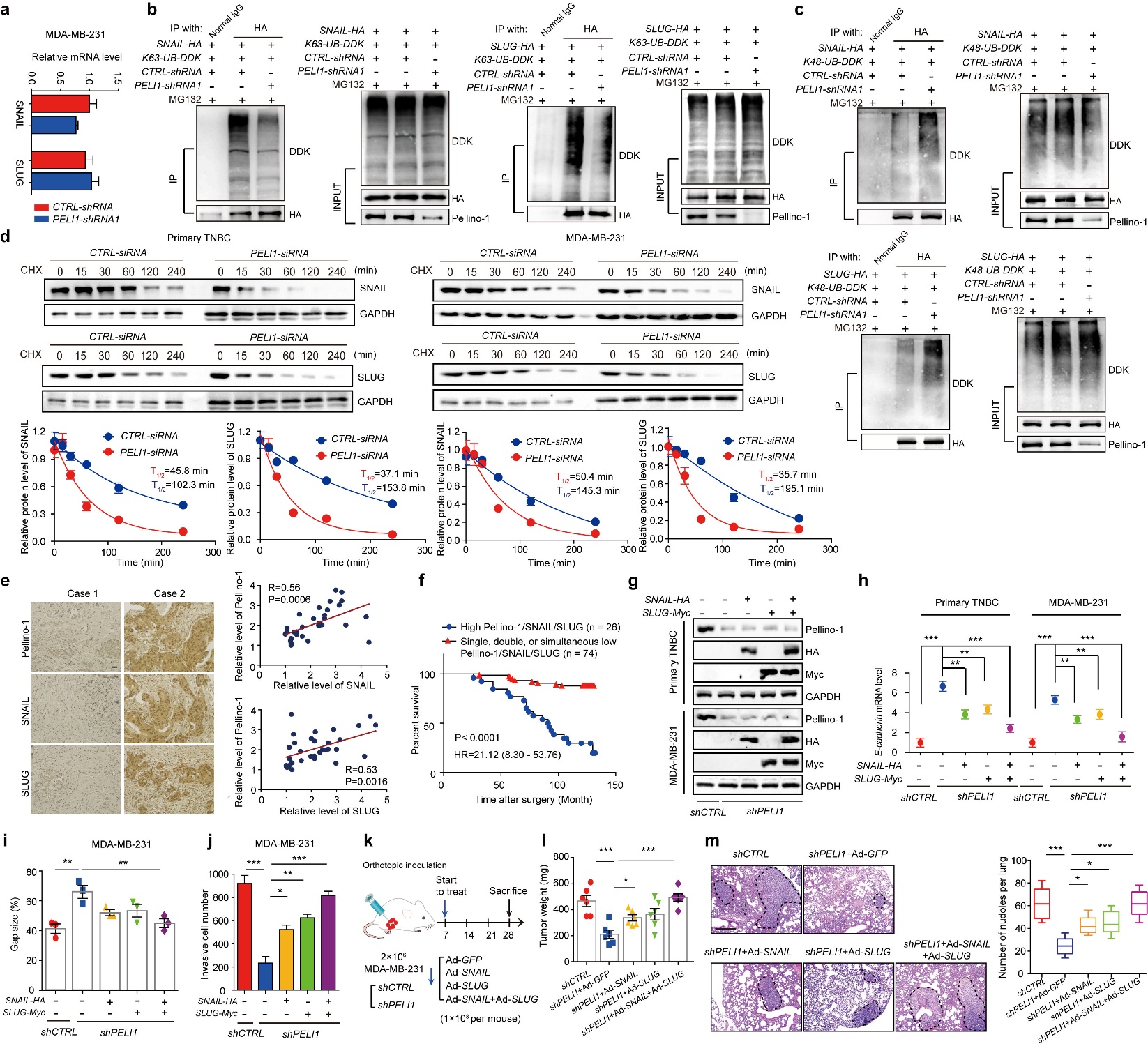
**

**Figure S2. Silencing *PELI1* suppresses TNBC through increasing SNAIL and SLUG degradation. a** PCR analyses of the mRNA level of SNAIL/SLUG after *PELI1* depletion (n = 3). **b** IP assay analyses of K63-mediated polyubiquitination of SNAIL and SLUG in MDA-MB-231 cells after *PELI1* knockdown (n = 3). **c** IP assay analyses of K48-mediated polyubiquitination of SNAIL and SLUG in MDA-MB-231 cells after *PELI1* knockdown (n = 3). **d** Quantitative analyses of SNAIL and SLUG degradation in the presence of CHX (10 μg/mL) in primary TNBC cells and MDA-MB-231 cells after Pellino-1 knockdown. The half-lives (t_1/2_) of SNAIL and SLUG degradation were calculated by one phase decay analysis (n = 3). **e** Representative IHC images for Pellino-1, SNAIL and SLUG expression from breast cancer patients who showed little expression for Pellino-1, SNAIL, and SLUG (left) versus those showing strong expression for those three proteins (right) are shown. Scale bar, 200 μm. And correlation between Pellino-1 and SNAIL, or Pellino-1 and SLUG expression in TNBC patients (n = 33). Each data point represents the value from an individual patient. Statistical significance was measured by Pearson’s correlation test. **f** Kaplan–Meier plot of overall survival of patients with breast cancer stratified by Pellino-1, SNAIL, and SLUG co-expression level. Patients were divided into 2 groups: high Pellino-1/SNAIL/SLUG expressions vs single, double, or simultaneous low Pellino-1/SNAIL/SLUG expression. **g** Immunoblots were used to confirm Pellino-1 knockdown and SNAIL and SLUG overexpression in cells with indicated treatment (n = 3). **h** Quantitative analyses of *E-cadherin* mRNA level by PCR assay in *PELI1*-silenced primary TNBC cells and MDA-MB-231 cells after overexpression of SNAIL, SLUG or both (n = 3). **i** Wound healing assay showing that overexpression of SNAIL or SLUG recovers the Pellino-1 depletion-inhibited invasion and migration in MDA-MB-231 cells (n = 3). **j** Quantitative analyses of transwell assay showing that enhanced SNAIL or SLUG expression recovers invasion suppressed by *PELI1* knockdown (n = 3). **k** The strategy for studying the anti-tumor effects of *PELI1* knockdown in the presence of overexpressed SNAIL, SLUG or both *in vivo*. **l, m** MDA-MB-231 cells transfected with either *CTRL*-*shRNA* or *PELI1-shRNA* were injected into mammary fat pad of nude mice unilaterally. One week later, mice were infected with Ad-*GFP*, Ad-*SNAIL*, or Ad-*SLUG* for 3 weeks (n = 6 per group). Data are statistical analyses of the tumor weights (l), histopathologic images of lung metastatic nodules and statistical analyses of lung metastatic nodules (m) of indicated group. Scale bar, 200 μm. *P < 0.05, **P < 0.01, ***P < 0.001.

**Figure. S3.**


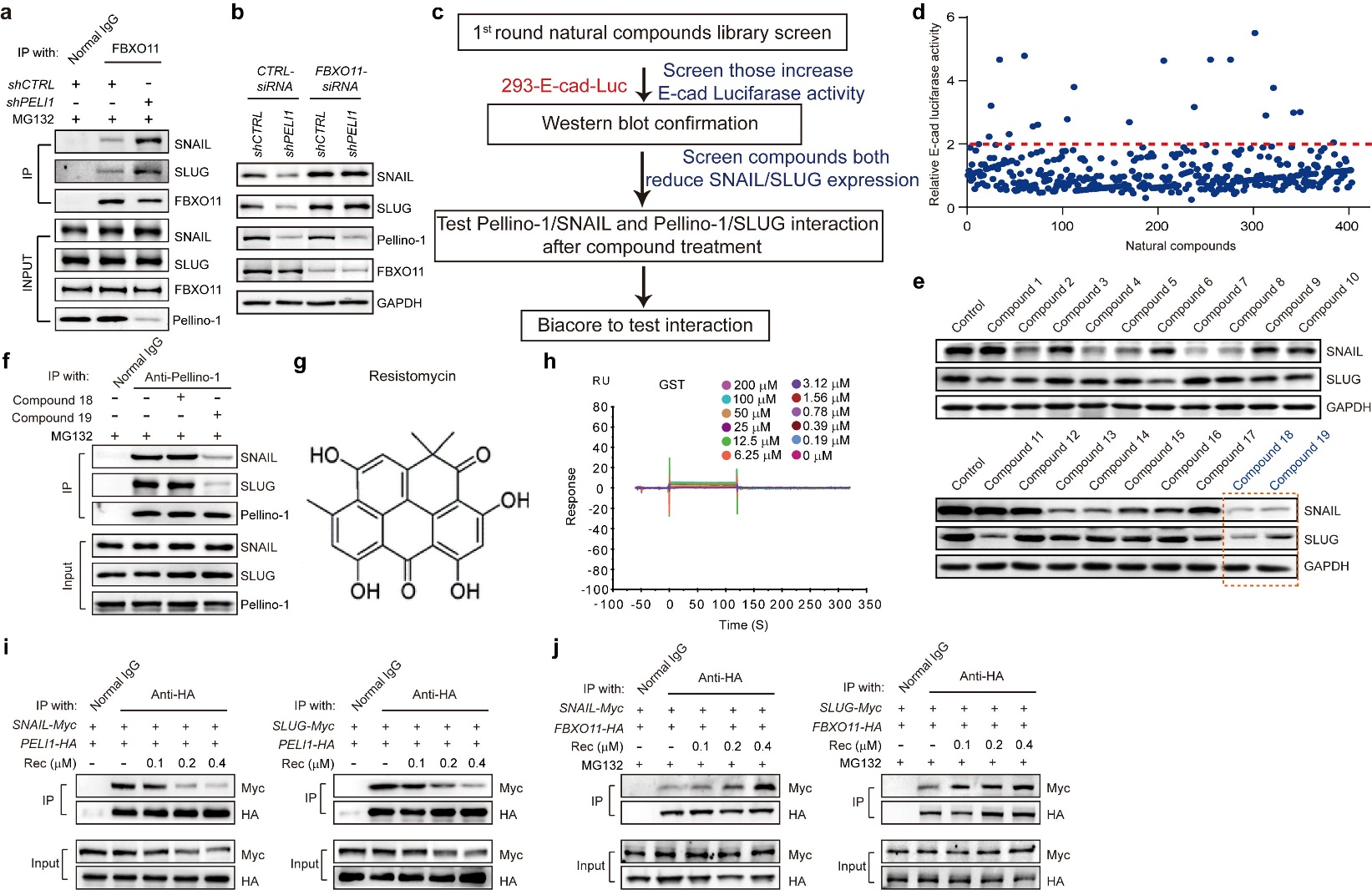


**Figure S3. Resistomycin binds to and disturbs Pellino-1 interaction with SNAIL/SLUG.** **a** IP assay analyses showing the FBXO11/SNAIL or FBXO11/SLUG interaction in MDA-MB-231 cells after *PELI1* knockdown (n = 3). **b** Western blot showing the expression of SNAIL and SLUG in FBXO11-silenced MDA-MB-231 cells after Pellino-1 knockdown (n = 3). **c** Experimental procedure flow chart for identification of natural compound(s) inhibiting Pellino-1. Individual product was exposed to 293-E-cadherin-Luc cells for 24 h. Positive product hits, which could result in more than 2-fold increase of luciferase activity of E-cadherin, were then examined by immunoblot analysis to identify candidates which could reduce protein level of SNAIL and SLUG simultaneously. Then Co-IP assay were performed to determine whether they could disrupt Pellino-1 and SNAIL/SLUG interaction for further confirmation. Finally, only the compounds that interacted with Pellino-1 were considered as candidate inhibitors of Pellino-1 and selected for functional assays. **d** Luciferase based natural product library screen identified multiple candidates, when added to 293-E-cadherin-Luc cells, increased luciferase activity by more than 2 fold (n = 3). **e** Sample blot showing the expression of SNAIL and SLUG after compounds treatment (n = 3). **f** IP assay analyses showing the effect of candidates on the interaction between Pellino-1 and SNAIL/SLUG in primary TNBC cells (n = 3). **g** Chemical structure of Resistomycin. **h** The indicated concentrations of Resistomycin were passed over immobilized GST on CM5 sensor chips and the kinetic interaction of Resistomycin with GST was determined with SPR analyses (n = 3). **i** Sample blots showing the effect of Resistomycin on SNAIL/Pellino-1 interaction and SLUG/Pellino-1 interaction in primary TNBC cells (n = 3). **j** IP assay analyses showing the FBXO11/SNAIL or FBXO11/SLUG interaction after Resistomycin treatment (n = 3).

**Figure. S4.**


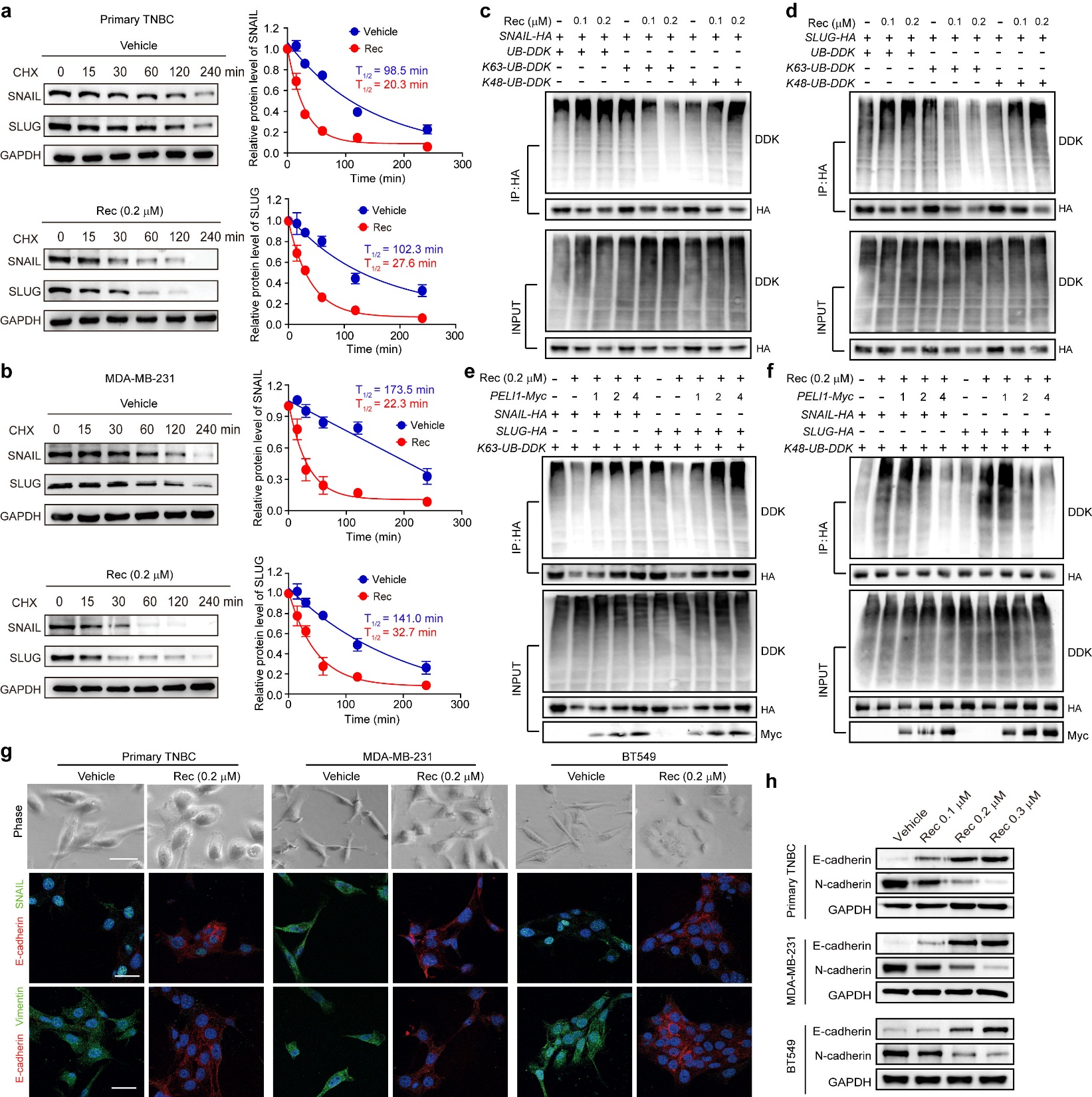


**Fig. S4 Resistomycin reverses EMT by reducing the SNAIL/SLUG expression in TNBC cells**. **a, b** Quantitative analyses of SNAIL and SLUG degradation in the presence of CHX (10 μg/mL) in primary TNBC cells (**a**) and MDA-MB-231 cells (**b**) after Resistomycin treatment. The half-lives (t_1/2_) of SNAIL and SLUG degradation were calculated by one phase decay analysis (n = 3). GAPDH was used as a loading control for IB. **c, d** IP assay analyses of total, K48-, or K63- mediated polyubiquitination of SNAIL (c) and SLUG (d) in MDA-MB-231 cells after Resistomycin treatment (n = 3). **e, f** IP assay analyses of K63- (e), or K48- (f) mediated polyubiquitination of SNAIL and SLUG in Resistomycin treated MDA-MB-231 cells after *PELI1* overexpression (n = 3). **g** Phase contrast and EMT markers immunofluorescence images of primary TNBC, MDA-MB-231 and BT549 cells treated with the indicated concentrations of Resistomycin for 24 h (n = 3). Scale bars, 100 μm for bright field and 150 μm for E-cadherin and Vimentin. **h** Sample immunoblots of the expression of EMT markers in primary TNBC cells, MDA-MB-231 and BT549 cells in the presence of various concentrations of Resistomycin (n = 3). GAPDH was used as a loading control for immunoblotting.

**Figure. S5.**

**
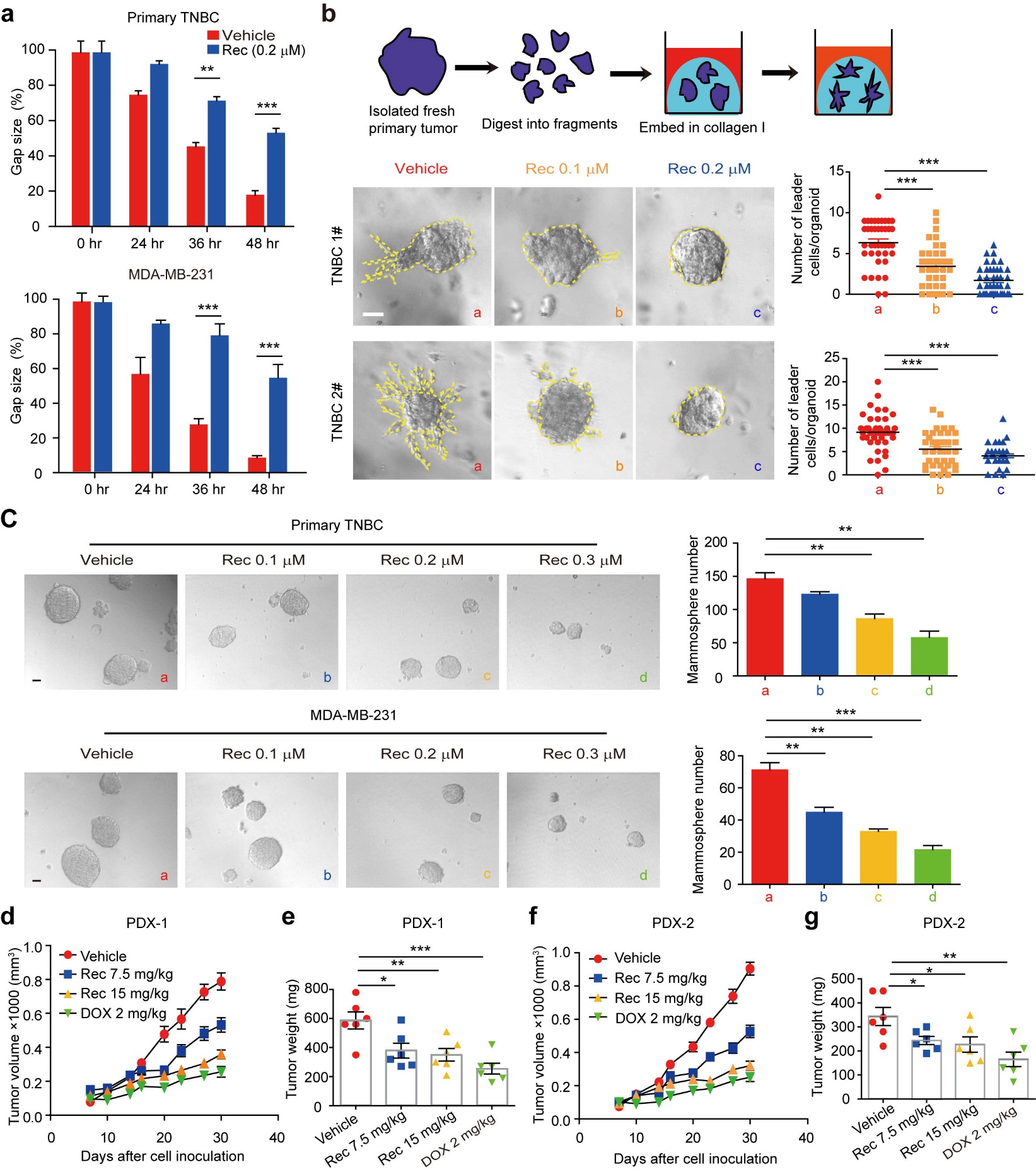
**

**Figure S5. Inhibition of Pellino-1 by Resistomycin attenuates TNBC invasion and migration. a** Primary TNBC and MDA-MB-231 monolayers were scraped by a sterile micropipette tip and the cells were treated with various concentrations of Resistomycin for 24 h, 36 h, and 48 h. The residual gap between the migrating cells from the opposing wound edge is expressed as a percentage of the initial scraped area (n = 3). **b** Tumor organoids were used to evaluate the inhibitory effect of Resistomycin on breast cancer invasion (n = 3). The primary TNBC tumors are digested to tumor organoids, and embedded in 3D collagen I matrix. Protrusive leader cells are readily identiﬁed as invasive strands. Scale bar, 50 μm. **c** Tumor spheroid formation of primary TNBC and MDA-MB-231 cells treated with various concentrations of Resistomycin (n = 3). Data were showed as number of mammospheres per 1000 cells. Scale bar, 50 μm. **d-g** The anti-tumor effects of Resistomycin on patient-derived tumor xenograft (PDX) models from TNBC patients were investigated. Doxorubicin (DOX) was used as a positive control. Tumors were injected into mammary fat pad of NSG mice unilaterally. One week later, the mice were treated with Vehicle, Resistomycin, or Doxorubicin for 3 weeks (n = 6 per group). The data are the statistical analyses of tumor growth curves (d, f) and the tumor weights (e, g). *P < 0.05, **P < 0.01, ***P < 0.001.

**Figure. S6.**

**
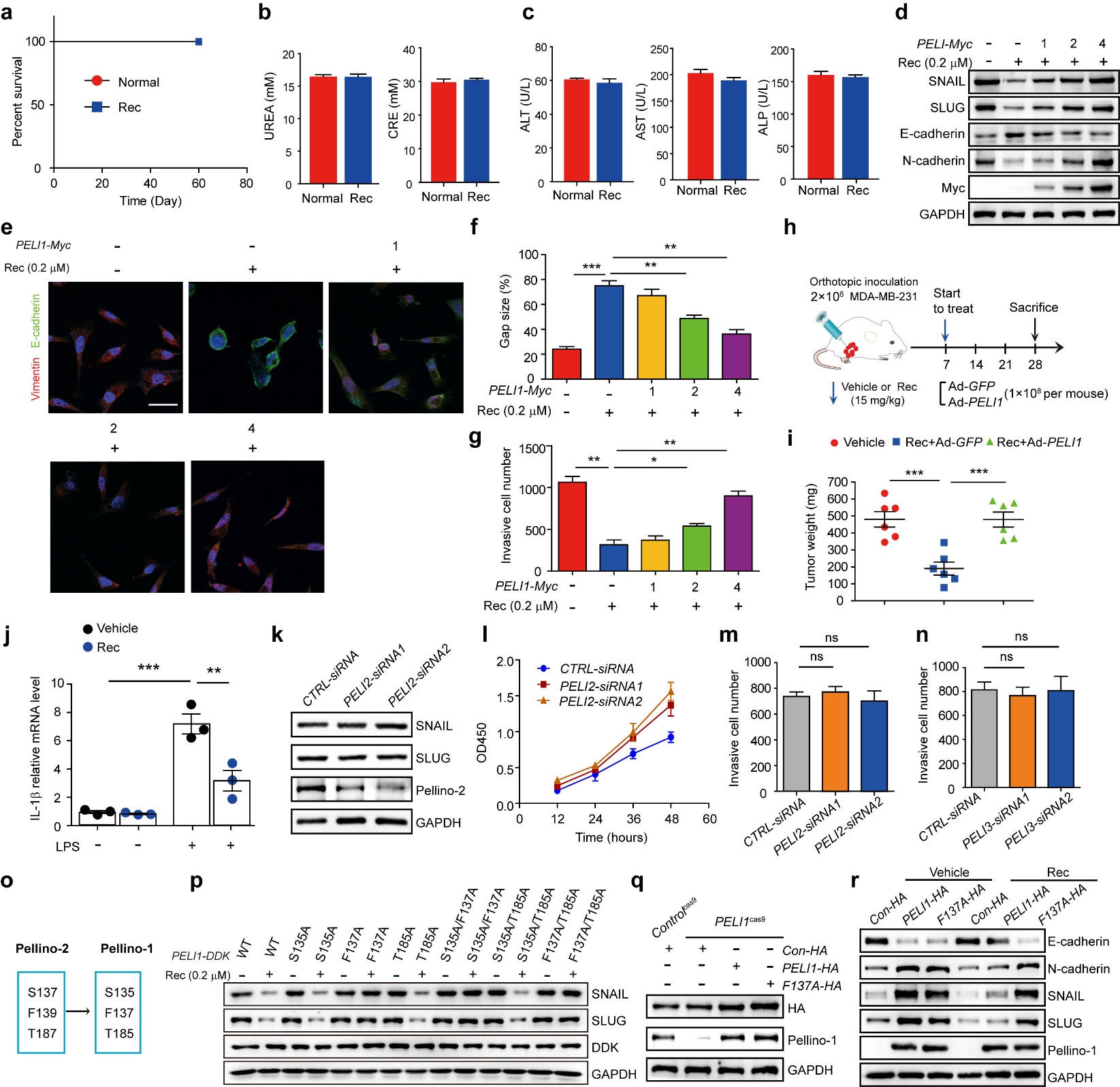
**

**Figure S6. Resistomycin inhibits TNBC by binding to F137 of Pellino-1. a-c** NOD-SCID mice were i.p. injected with or without Resistomycin (15 mg/kg), twice a week for 2 months. At the end of 2 months, the living mice were processed (n = 6 per group). Survival rates of indicated mice were analyzed by Kaplan-Meier (a). The serum UREA and CRE levels of normal and Resistomycin-treated mice were detected to evaluate renal function (b). The serum ALT, AST and ALP levels of normal and Resistomycin-treated mice were detected to evaluate liver function (n = 6 per group) (c). **d** Sample immunoblots of EMT markers in the presence of Resistomycin in *PELI1*-overexpressed MDA-MB-231 cells (n = 3). GAPDH was used as a loading control for IB. **e** Immunofluorescence images of EMT markers in MDA-MB-231 cells after indicated treatment (n = 3). Scale bar, 150 μm. **f, g** Quantitative analyses of wound healing assay (f) and transwell assay (g) showing that enhancing Pellino-1 expression recovers MDA-MB-231 cells migration and invasion which were inhibited by Resistomycin (n = 3). **h** The strategy for studying the anti-tumor effects of Resistomycin in the presence or absence of Pellino-1 overexpression *in vivo*. **i** MDA-MB-231 cells were injected into mammary fat pad of nude mice unilaterally. One week later, mice were treated with vehicle or Resistomycin, and infected with Ad-*GFP* or Ad-*PELI1* for 3 weeks (n = 6 per group). Data are statistical analyses of the tumor weights. **j** Quantitative RT-PCR analyses of *IL-1β* mRNA level in RAW264.7 cells stimulated with 100 ng/ml LPS for 24 h with or without further treatment with Rec for 12 h (n = 3). **k** Silencing *PELI2* did not change the expression of SNAIL or SLUG in MDA-MB-231 cells (n = 3). **l** Silencing *PELI2* showed no effect on the proliferation of MDA-MB-231 cells (n = 3). **m** Silencing *PELI2* showed no effect on the invasion of MDA-MB-231 cells (n = 3). **n** Silencing *PELI3* did not effect the invasion of MDA-MB-231 cells (n = 3). **o** Schematic diagram of Pellino-1 point mutants. **p** Sample blot showing SNAIL or SLUG expression in wild type *PELI1*-transfected or *PELI1* mutants-transfected MDA-MB-231 cells in the presence of Resistomycin (n = 3). **q** *Control^Cas9^* and *PELI1^Cas9^* MDA-MB-231 cells were transfected with indicated plasmids. After 24 h of transfection, cell lysates from these transfectants were immunoblotted with indicated antibody (n = 3). **r** Sample blot showing the effect of Resistomycin on EMT markers of indicated xenograft tumors (n = 3). *P < 0.05, **P < 0.01, ***P < 0.001.

**Table S1.**

**Supplementary Table 1.** Clinical information of breast cancer patients. Related to Fig. 1a.

| No. | Age | Grade | TNM | IHC staining |
| --- | --- | --- | --- | --- |
| Luminal-1 | 41 | G3 | 100 | Ki67(+), Cerb B-2(+), ER(+), PR(-), P53(-) |
| Luminal-2 | 53 | G2 | 200 | Ki67(+), Cerb B-2(+), ER(+), PR(-), P53(+) |
| Luminal-3 | 65 | G2 | 110 | Ki67(+), Cerb B-2(+), ER(+), PR(-), P53(+) |
| Luminal-4 | 74 | G2 | 200 | Ki67(+), Cerb B-2(-), ER(+), PR(+), P53(+) |
| Luminal-5 | 48 | G2 | 110 | Ki67(+), Cerb B-2(+), ER(+), PR(+), P53(+) |
| Luminal-6 | 62 | G2 | 110 | Ki67(+), Cerb B-2(+), ER(+), PR(+), P53(+) |
| Luminal-7 | 68 | G2 | 210 | Ki67(+), Cerb B-2(+), ER(+), PR(+), P53(-) |
| Luminal-8 | 71 | G2 | 110 | Ki67(-), Cerb B-2(+), ER(+), PR(+), P53(+) |
| Luminal-9 | 41 | G1 | 100 | Ki67(+), Cerb B-2(+), ER(+), PR(+), P53(+) |
| HER2^+^-1 | 54 | G3 | 100 | Ki67(+), Cerb B-2(+), ER(-), PR(-), P53(+) |
| HER2^+^-2 | 54 | G2 | 200 | Ki67(+), Cerb B-2(+), ER(-), PR(-), P53(-) |
| HER2^+^-3 | 47 | G2 | 110 | Ki67(+), Cerb B-2(+), ER(-), PR(-), P53(+) |
| HER2^+^-4 | 53 | G3 | 200 | Ki67(+), Cerb B-2(+), ER(-), PR(-), P53(+) |
| HER2^+^-5 | 70 | G2 | 200 | Ki67(+), Cerb B-2(+), ER(-), PR(-), P53(-) |
| HER2^+^-6 | 36 | G2 | 210 | Ki67(+), Cerb B-2(+), ER(-), PR(-), P53(-) |
| HER2^+^-7 | 69 | G2 | 220 | Ki67(+), Cerb B-2(+), ER(-), PR(-), P53(+) |
| HER2^+^-8 | 54 | G2 | 200 | Ki67(+), Cerb B-2(+), ER(-), PR(-), P53(+) |
| HER2^+^-9 | 46 | G2 | 220 | Ki67(+), Cerb B-2(+), ER(-), PR(-), P53(+) |
| Basal-like-1 | 41 | G3 | 200 | Ki67(+), Cerb B-2(-), ER(-), PR(-), P53(+) |
| Basal-like-2 | 63 | G3 | 320 | Ki67(+), Cerb B-2(-), ER(-), PR(-), P53(+) |
| Basal-like-3 | 41 | G3 | 100 | Ki67(+), Cerb B-2(-), ER(-), PR(-), P53(-) |
| Basal-like-4 | 57 | G2 | 200 | Ki67(+), Cerb B-2(-), ER(-), PR(-), P53(+) |
| Basal-like-5 | 55 | G2-G3 | 110 | Ki67(+), Cerb B-2(-), ER(-), PR(-), P53(+) |
| Basal-like-6 | 53 | G3 | 220 | Ki67(+), Cerb B-2(-), ER(-), PR(-), P53(+) |
| Basal-like-7 | 63 | G3 | 100 | Ki67(+), Cerb B-2(-), ER(-), PR(-), P53(-) |
| Basal-like-8 | 29 | G3 | 100 | Ki67(+), Cerb B-2(-), ER(-), PR(-), P53(-) |
| Basal-like-9 | 57 | G3 | 100 | Ki67(+), Cerb B-2(-), ER(-), PR(-), P53(-) |
| Normal-1 | Adjacent non-tumor tissue for Luminal-1 | | | |
| Normal-2 | Adjacent non-tumor tissue for HER2^+^-1 | | | |
| Normal-3 | Adjacent non-tumor tissue for Basal-like-1 | | | |
| Normal-4 | Adjacent non-tumor tissue for Luminal-4 | | | |
| Normal-5 | Adjacent non-tumor tissue for HER2^+^-4 | | | |
| Normal-6 | Adjacent non-tumor tissue for Basal-like-4 | | | |
| Normal-7 | Adjacent non-tumor tissue for Luminal-7 | | | |
| Normal-8 | Adjacent non-tumor tissue for HER2^+^-7 | | | |
| Normal-9 | Adjacent non-tumor tissue for Basal-like-7 | | | |

**Table S2.**

**Supplementary Table 2** Patient-related information. Related to Fig.1c, Fig. S5b and Fig. S5d-g.

ID Age Sex Tumor Site Cancer Type IHC Staining

Patient 1 51 Femal Breast Triple-negative ER(-),PR(-),HR(-)

Patient 2 60 Femal Breast Triple-negative ER(-),PR(-),HR(-)
